# Consistently High Unprotected Anal Intercourse (UAI) and factors correlated with UAI among men who have sex with men: implication of a serial cross-sectional study in Guangzhou, China

**DOI:** 10.1186/s12879-014-0696-8

**Published:** 2014-12-18

**Authors:** Weibin Cheng, Weiming Tang, Fei Zhong, Giridhar R Babu, Zhigang Han, Faju Qin, Kai Gao, Huixia Mai, Yuteng Zhao, Caiyun Liang, Lirui Fan, Hao Wu, Huifang Xu, Ming Wang

**Affiliations:** Department of AIDS/STD Control and Prevention, Guangzhou Center for Disease Control and Prevention, No.1, Qide Road, Jiahe, Baiyun District, Guangzhou, Guangdong Province, 510440 China; University of North Carolina Project-China, No.2 Lujing Road, Yuexiu District, Guangzhou, Guangdong Province, 510095 China; School of Public Health, Sun Yat-Sen University, No.74 2nd Yat-Sen Road, Yuexiu District, Guangzhou, Guangdong, Province, 510080 China; Department of Epidemiology, Public Health Foundation of India, IIPH H, Bangalore Campus, SIHFW Premises, 1st cross, Magadi road, Bangalore, Karnataka, 560023 India

**Keywords:** Unprotected anal intercourse (UAI), Men who have sex with men (MSM), Human immunodeficiency virus (HIV), Trend, Serial cross-sectional study, China

## Abstract

**Background:**

China experiencing an increasing HIV epidemic among men who have sex with men (MSM), and unprotected anal intercourse (UAI) has played a key role in this process. The aims of this study were to examine the trend of UAI and to explore the factors correlated with UAI among MSM in Guangzhou, China.

**Methods:**

Data from 2008 to 2013 were retrieved from the annual serological and behavioral surveys system. We collected information on demographic, HIV related sexual behavior with men and women, access to HIV prevention services, and symptoms of sexually transmitted infections. Chi-square test was used to examine the similarity of the participants during the study period. Univariate and multivariate logistic regression were conducted to test the factors associated with UAI. Trend test was used to check the change of UAI in different characteristic stratums during the study period.

**Results:**

In total, 58.4% (range from 54.5% to 62.0%) of the participants reported that they engaged in UAI in the past six months. Participants who aged less than 20 [Adjusted Odds Ratio (AOR): 2.22, 95% Confidential Interval (CI): 1.07-4.61], only attended elementary school (or less) (AOR: 1.41, 95% CI: 1.04-1.90), cohabiting with male partner (AOR: 2.15, 95% CI: 1.66-2.79), divorced or widowed (AOR: 2.80, 95% CI: 1.54-5.07), did not test for HIV in the past year (AOR: 1.36, 95% CI: 1.12-1.65), and had 10 or more partners in the past six months (AOR: 1.85, 95% CI: 1.18-2.91) had higher odds of UAI. However, the proportions of UAI were stable in different stratums during the study period.

**Conclusions:**

The proportion of MSM engaged in UAI was consistently high during the study period. Effective intervention strategies, which include but not limit to risk reduction counseling and testing services, are urgently needed to bring down the risk behaviors of the MSM in Guangzhou, in order to control HIV/STIs epidemic in this specific population.

## Background

Generally, the Chinese society ascribes negative connotation to homosexual as aberrant behavior [1]. Men who have sex with men (MSM) continue to lead clandestine risk behaviors while they continue to stay married and have children [2]. Even lots of effort already put, we still confront increasing HIV/AIDS epidemic among MSM [3],[4], while other high risk groups have been met with greater success [5]. It is estimated that there are nearly 10 million MSM in China [3],[4],[6]. The raising epidemic of HIV and sexually transmitted infections (STIs) may further worsen the burden of HIV/STIs in China [7],[8]. Targeting intervention could bring down this burden, and there is also evidence available on different sexual risk behavior among MSM [4]. It is estimated that the proportion of MSM engaging in high-risk sexual behavior is changing over a period of time [9]. However, a clear pattern of these changes is yet to be studied in different geographical areas in China.

It is recognized that MSM have high proportion of risky behaviors such as unprotected sex, drug use, and several adverse health outcomes [10]. The enormity of the HIV epidemic warrants for studies and interventions targeting MSM. Our earlier study indicated that unprotected anal intercourse (UAI) significantly decreased as result of risk reduction counseling [4]. However, it is unclear whether such change in behavior was confounded by other intervention efforts.

Mostly, MSM are treated as a homogenous group and therefore the varied risk propensity based on contextual factors are often ignored [11]. UAI is consider to be the key reason of HIV and other STIs transmission among MSM of China [10]. Therefore, it is important to explore the contextual determinants of UAI, as they will provide tools for detection of potential HIV transmission.

Guangzhou is the capital and largest city of Guangdong province; it is also the home 12.78 million people. A serial cross-sectional study was conducted between 2008 and 2013 in Guangzhou, China. HIV risk-reduction counseling and testing was implemented for each participant during each visit of the participants. This counseling procedure included pre- and post-test counseling services. The objectives of the study were to explore the risk behaviors and determinants of HIV/syphilis acquisition in Guangzhou, and assessed the effectiveness of a counseling session in reducing the risk behaviors of MSM.

## Methods

### Materials and methods

The current reported serial cross-sectional study was conducted between 2008 and 2013 in Guangzhou, China.

### Sample size

In each year of the study period, in order to observe the behaviors of MSM in Guangzhou, China, a certain number of MSM were recruited. The sample size that we needed at each year was estimated based on the following formula [12], by using the known HIV epidemic of Guangzhou:

$$n=\;\left[\mathrm{DEFF}*\mathrm{Np}\left(1-p\right)\right]/[\left(d^{2}/{Z^{2}}_{1-\alpha /2}*\left(N-1\right)+p*\left(1-p\right)\right)$$

From our previous data (unpublished, using the method of capture-recapture), we estimated that there were about 35,000 MSM in Guangzhou at the year of 2008. By using HIV prevalence of 5.2% in 2008 [13], confidence limit (d) of 5%, a design effect (DEFF) of 2, and an alpha (α) of 0.05, a total of 152 MSM were needed. However, in order to increase the power of our study, we decided to increase the sample size to 400, instead of 152. In 2013, Guangzhou was selected as one of the MSM incidence study sites by National Center for AIDS/STD Control and Prevention, and the program need at least 600 MSM, thus we further recruited 200 more participants and included them into our current reported study, in order to further increase the power of our study.

### Participant recruitment

Our study was conducted between April and July of each year between the years of 2008 and 2013. In order to be comfortable and convenient to the participants, our study was conducted within office site of a local gay community (Guangzhou Tongzhi). This site was supported by Guangzhou Center for Disease Control and Prevention (CDC), and it was listed as one HIV voluntary counseling and testing site of Guangzhou city. During the study period, the same interviewers conducted the survey by following the same study protocol which developed by Guangzhou CDC. This site is the main site that targeting MSM in Guangzhou, and it tested about 6000 to 8000 MSM annually, which accounted for about 90% of the MSM tested in Guangzhou at each year. In order to meet the inclusion criteria, the participants must be male, had anal or oral sex with men in the last 12 months, at least 18 years old and had lived in Guangzhou for at least 6 months before our study. In order to increase the representative of our study, snow-ball sampling method was used to recruit the eligible participants at each year. To encourage the participation of eligible MSM, incentive (A gift of 2 US dollars and cash about 3 US dollars) was given to each participated MSM.

### Behavioral Measures

Face-to-face interview was used to collect information from participants after the blood drawing. Information was collected regarding details on demographic characteristics, knowledge and attitudes about HIV, preventive services, recent sexual behaviors and drug use of the participants. Coverage of HIV intervention was defined as the percentage of participants who received any kind of HIV related service from CDC in the past one year. In our study, UAI was defined as inconsistent use of condoms during anal sex with male partners in the past six months. UVI was defined as inconsistent use of condoms during vaginal sex with female partners in the past six months.

We offered HIV risk-reduction counseling and testing for each participant, which included pre- and post-test counseling services. If the participants were HIV positive, he would be referred to the Chinese HIV/AIDS Care program. If the participants were syphilis positive, they will be referred to the STIs clinics at Guangzhou for syphilis treatment.

The ethics Committee, Guangzhou CDC reviewed and approved the study protocol. All participants were provided written informed consent, and had the right to withdraw from the study at any time.

### Serologic measures

Subsequent to signing the written informed consent, 5 ml intravenous blood was drawn from each participant ahead of the interview. HIV screening was done by using enzyme-linked immunoassays (ELISAs; Diagnostic Kit for Antibody to Human Immunodeficiency Virus, BioMérieux, Boxtel, The Netherlands, Beijing BGI-GBI Biotech, Beijing, China). After screened to be HIV positive by ELISA method, confirmation was done by Western Blot (WB;MP Biomedicals Asia Pacific Pte Ltd, Singapore). Syphilis screening was performed by rapid plasma reagents (RPR; Shanghai Kehua Bioengineering Co. Ltd, Shanghai, China). Specimens tested positive by RPR were confirmed by the Treponema Pallidum particle agglutination test (TPPA; Livzon Group Reagent Factory, Zhuhai, China). In our study, syphilis positive was defined as both RPR and TPPA positive.

### Data analysis

Data was double entered and cleaned by using EpiData (version 3.1, Denmark) and Microsoft excel. Statistical analysis was performed by using IBM SPSS Statistic for windows version 18 (SPSS Inc., Chicago, USA). Descriptive analysis was conducted to determine the distribution of the demographic factors and behaviors of the participants. Chi-square test was used to examine the similarity of the participants during the study period. Univariate and multivariate logistic regression were conducted to test the factors associated with UAI. Stepwise method was used in our multivariate logistic regression. The independent variable in the univariate model included age, marital status, Hukou (registered permanent residence), education level, ethnicity, monthly income, age at first sex with male, sexual orientation, venues for meeting partners, UVI, HIV testing history, and preventive intervention services received. The variables that had P-value less than 0.10 during the preliminary analysis were included in the multivariate model.

In order to examine the change of UAI within different strata of interesting factors, we further stratified the participants based on the characters that correlated with UAI from the logistic regression model. After the stratification, we calculated the percentage of UAI at each stratum in each year, and we further performed trend test to check whether the percentage of UAI was changed in different stratums during the study period. All statistical significance test results are reported as p-values, where less than 0.05 was used to define significance.

## Results

In our study, a total of 2603 participants were recruited between the year of 2008 and 2013. The total number of participant recruited annually gradually increased during the study period, even there was a lit bit of wane (Table 1).

**Table 1:** **Demographic characteristics and key behaviors of MSM in Guangzhou, China, 2008–2013**

Characteristics	2008	2009	2010	2011	2012	2013	Total	χ ^2^value	***p*** ^a^
	(N = 379)	(N = 385)	(N = 405)	(N = 400)	(N = 401)	(N = 633)	(N = 2603)		
	n (%)	n (%)	n (%)	n (%)	n (%)	n (%)	n (%)		
Age (years)								22.80	0.299
<20	14 (3.7)	10 (2.6)	13 (3.2)	15 (3.8)	9 (2.2)	15 (2.4)	76 (2.9)		
20-29	217 (57.3)	227 (59.0)	245 (60.5)	250 (62.5)	228 (56.9)	385 (60.8)	1552 (59.6)		
30-39	111 (29.3)	113 (29.4)	120 (29.6)	101 (25.3)	128 (31.9)	174 (27.5)	747 (28.7)		
40-49	35 (9.2)	27 (7.0)	23 (5.7)	25 (6.3)	24 (6.0)	49 (7.7)	183 (7.0)		
≥50	2 (0.5)	8 (2.1)	4 (1.0)	9 (2.3)	12 (3.0)	10 (1.6)	45 (1.7)		
Marital status								40.73	<0.001
Single	214 (54.5)	232 (60.3)	256 (63.2)	260 (65.0)	258 (64.3)	430 (67.9)	1650 (63.4)		
Cohabiting with male partner	67 (17.7)	69 (17.9)	62 (15.3)	46 (11.5)	63 (15.7)	104 (16.4)	411 (15.8)		
Married	79 (20.8)	69 (17.9)	74 (18.3)	89 (22.3)	69 (17.2)	76 (12.0)	456 (17.5)		
Divorced or widowed	19 (5.0)	15 (3.9)	13 (3.2)	5 (1.3)	11 (2.7)	23 (3.6)	86 (3.3)		
Hukou (registered permanent residence)								57.46	<0.001
Guangzhou	105 (27.7)	122 (31.7)	122 (30.1)	115 (28.8)	134 (33.4)	235 (37.1)	833 (32.0)		
Other city in Guangdong province	85 (22.4)	84 (21.8)	90 (22.2)	67 (16.8)	94 (23.4)	186 (29.4)	606 (23.3)		
Outside Guangdong province	189 (49.9)	179 (46.5)	193 (47.7)	218 (54.5)	173 (43.1)	212 (33.5)	1164 (44.7)		
Han nationality	362 (95.5)	369 (95.8)	382 (94.3)	381 (95.3)	389 (97.0)	614 (97.0)	2497 (95.9)	6.37	0.272
Education level								64.16	<0.001^c^
Junior high school or lower	72 (19.0)	47 (12.2)	66 (16.3)	53 (13.3)	49 (12.2)	35 (5.5)	322 (12.4)		
Senior high school	111 (29.3)	99 (25.7)	115 (28.4)	140 (35.0)	103 (25.7)	110 (17.4)	678(26.0)		
College or higher	196 (51.7)	239 (62.1)	224 (55.3)	207 (51.8)	249 (62.1)	488 (77.1)	1603(61.6)		
Monthly income (CNY)								131.60	<0.001^c^
0	49 (12.9)	54 (14.0)	46 (11.4)	39 (9.8)	56 (14.0)	84 (13.3)	328 (12.6)		
≤2000	125 (33.0)	88 (22.8)	99 (24.5)	65 (16.3)	27 (6.7)	47 (7.4)	451 (17.3)		
2001-3000	85 (22.4)	95 (24.7)	94 (23.2)	112 (28.0)	78 (19.5)	98 (15.5)	562 (21.6)		
3001-4000	60 (15.8)	60 (15.6)	69 (17.0)	86 (21.5)	72 (18.0)	112 (17.7)	459 (17.6)		
>4000	60 (15.8)	88 (22.9)	97 (24.0)	98 (24.5)	168 (41.9)	292 (46.1)	803 (30.8)		
Self-perceived sexual orientation								26.23	0.003
Homosexual	232 (61.2)	247 (64.2)	264 (65.2)	260 (65.0)	247 (61.6)	451 (71.2)	1701 (65.3)		
Bisexual	106 (28.0)	109 (28.3)	117 (28.9)	120 (30.0)	119 (29.7)	148 (23.4)	719 (27.6)		
Uncertain	41 (10.8)	29 (7.5)	24 (5.9)	20 (5.0)	35 (8.7)	34 (5.4)	183 (7.0)		
Venues for meeting partners								170.40	<0.001
Bar, disco, tea house or club	28 (7.4)	31 (8.1)	25 (6.2)	27 (6.8)	22 (5.5)	22 (3.5)	155 (6.0)		
Bath house, sauna or massage	25 (6.6)	15 (3.9)	14 (3.5)	9 (2.3)	6 (1.5)	8 (1.3)	77 (3.0)		
Park, public toilet	57 (15.0)	25 (6.5)	24 (5.9)	18 (4.5)	40 (10.0)	5 (0.8)	169 (6.5)		
Internet	247 (65.2)	273 (70.9)	298 (73.6)	309 (77.3)	282 (70.3)	564 (89.1)	1973 (75.8)		
Other	22 (5.8)	41 (10.6)	44 (10.9)	37 (9.3)	51 (12.7)	34 (5.4)	229 (8.8)		
Age of first sex with male (years)								1.97	0.161^c^
<19	66 (17.4)	88 (22.9)	63 (15.6)	62 (15.5)	70 (17.5)	118 (18.6)	467 (17.9)		
19-24	195 (51.5)	178 (46.2)	181 (44.7)	179 (44.8)	192 (48.0)	364 (57.5)	1289 (49.5)		
≥25	118 (31.1)	119 (30.9)	161 (39.8)	159 (39.8)	138 (34.5)	151 (23.9)	846 (32.5)		
Number of male partners in the past 6 months								7.45	0.006^c^
1	94 (28.0)	123 (35.3)	121 (33.6)	123 (33.3)	136 (37.5)	184 (32.2)	781 (33.3)		
2	80 (23.8)	92 (26.4)	96 (26.7)	98 (26.6)	97 (26.7)	159 (27.8)	622 (26.5)		
3	69 (20.5)	66 (19.0)	63 (17.5)	66 (17.9)	72 (19.8)	112 (19.6)	448 (19.1)		
4-9	69 (20.5)	38 (10.9)	60 (16.7)	68 (18.4)	40 (11.1)	105 (18.4)	380 (16.2)		
≥10	24 (7.1)	29 (8.3)	20 (5.6)	14 (3.8)	18 (5.0)	12 (2.1)	117 (5.0)		
Had sex with female in the past six months	82 (21.6)	81 (21.0)	80 (19.8)	74 (18.5)	87 (21.7)	76 (12.0)	480 (18.4)	25.02	<0.001
UVI^b^	58 (15.3)	49 (12.7)	64 (15.8)	52 (13.0)	60 (15.0)	53 (8.4)	336 (12.9)	18.05	0.003
UAI^b^	233 (61.5)	210 (54.5)	240 (59.3)	248 (62.0)	227 (56.6)	361 (57.0)	1519 (58.4)	7.10	0.213
HIV test in the past 12 months	62 (16.4)	274 (71.2)	178 (44.0)	177 (44.7)	198 (49.7)	338 (53.4)	1227 (47.3)	247.15	<0.001
Coverage of HIV intervention	287 (75.7)	338 (87.8)	301 (74.3)	283 (70.8)	334 (83.3)	538 (85.0)	2081 (79.9)	60.94	<0.001

^a^Pearson chi-square test.

^b^UAI, unprotected anal intercourse; UVI, unprotected vaginal intercourse.

^c^Linear-by-linear association chi-square test.

### Study population characteristics and behaviors

Among the participants, more than three fifth were aged less than 30, and these percentages did not vary too much during the study period. About 63% of the participants reported that they were single. However, this percentage increased during the study period (54.5% in 2008, 60.3% in 2009, 63.2% in 2010, 65.0% in 2011, 64.3% in 2012 and 67.9% in 2013, P < 0.01). Our study also indicated that less than one third of the participants were residents of Guangzhou, even this percentage varied year by year (Table 1). About 62% of the participants received high education, and the proportion of the participants attended college or higher slightly increased during the study period (P < 0.01). In total, about 30% of the participants had monthly income less than RMB 2000 Yuan (US $320).

About two thirds of the participants admitted that their sexual orientation was homosexual, which ranged between 61.2% and 71.2% during the study period. In addition, about three quarters of the participants mainly found their sexual partners online, which was also varied year by year (65.2% in 2008, 70.9% in 2009, 73.6% in 2010, 77.3% in 2011, 70.3% in 2012 and 89.1% in 2013, P < 0.01). Table one also indicated that about 18% of the participants reported that they started to engage in sex male before 19 years old.

In our study, about one third of the participants reported that they only had one male partner in the past six months. This proportion also varied year by year (ranged between 28.0% in 2008 and 37.5 in 2012, P = 0.01). In addition, about 13% and 58% of the participants reported that they engaged in UVI and UAI in the past six months, respectively. About 47% (ranged between 16.4% and 71.2%, P < 0.01) of the participants tested for HIV in the past 12 months. In addition, about four fifth of the participants reported that they received HIV related intervention.

### Factors correlated with UAI

In our study, both univariate and multivariate model shown that compared with those participants who aged 40 or more, the participants aged less than 20 had significant higher odds of engaging in UAI (Crude Odds Ratio (COR) = 2.75, 95% CI:1.55-4.88, Adjusted Odds Ratio (AOR) = 2.22, 95% CI: 1.07-4.61). And the univariate model indicated that the risk of engaging in UAI increased with the decreasing of age (Table 2). Same phenomenon was also observed for education, with poorer education status of the participants associated with the higher odds of engaging in UAI. The adjusted model showed that the odds of the participants who only attended elementary school (or less) was about 1.41 (95% CI: 1.04-1.90) times of the odds of those attended college.

**Table 2:** **Factors associated with unprotected anal intercourse among MSM in Guangzhou, 2008–2013, by Logistic regression model** ^**a**^

Factor	UAI ^b^% (n)	***COR*** (95% CI) ^c^	***AOR*** (95% CI) ^d^
Age (years)			
<20	73.7 (56)	2.75 (1.55-4.88)^f^	2.22 (1.07-4.61)^e^
20-29	59.0 (916)	1.42 (1.07-1.87)^e^	1.30 (0.87-1.93)
30-39	57.8 (432)	1.35 (1.00-1.81)	1.17 (0.81-1.67)
≥40	50.4 (115)	1.00	1.00
Ethnicity			
Han	58.5 (1461)	1	-
Others	54.7 (58)	0.86 (0.58-1.27)	
Hukou (registered permanent residence)			
Guangzhou	56.7 (472)	1	-
Other city in Guangdong province	58.4 (354)	1.07 (0.87-1.33)	
Outside Guangdong province	59.5 (693)	1.13 (0.94-1.35)	
Education level			
Junior high school or lower	63.4 (204)	1.35 (1.06-1.73)^e^	1.41 (1.04-1.90)^e^
Senior high school	61.2 (415)	1.23 (1.03-1.48)^e^	1.23 (0.99-1.51)
College or higher	56.1 (900)	1.00	1.00
Marital status			
Single	54.7 (903)	1.00	1.00
Cohabiting with male partner	72.0 (296)	2.13 (1.68-2.70)^f^	2.15 (1.66-2.79)^f^
Married	56.8 (259)	1.08 (0.88-1.34)	1.28 (0.96-1.71)
Divorced or widowed	70.9 (61)	2.02 (1.26-3.25)^f^	2.80 (1.54-5.07)^f^
Monthly income (CNY)			
0	60.4 (198)	1	-
≤2000	62.5 (282)	1.10 (0.82-1.47)	
2001-3000	57.3 (322)	0.88 (0.67-1.16)	
3001-4000	57.3 (263)	0.88 (0.66-1.16)	
>4000	56.5 (454)	0.85 (0.66-1.11)	
Self-perceived sexual orientation			
Homosexual	59.5 (1012)	1	-
Bisexual	57.0 (470)	0.90 (0.76-1.08)	
Uncertain	53.0 (97)	0.77 (0.57-1.04)	
Venues for meeting partners			
Bar, disco, tea house or club	60.6 (94)	1	-
Bath house, sauna or massage	61.0 (47)	1.02 (.058-1.780	
Park, public toilet	55.4 (96)	0.81 (0.52-1.27)	
Internet	58.5 (1155)	0.92 (0.66-1.28)	
Other	56.3 (129)	0.84 (0.55-1.27)	
Age of first sex with male (years)			
<19	63.8 (298)	1.00	1.00
19-24	58.7 (756)	0.80 (0.65-1.00)	0.93 (0.73-1.20)
≥25	55.0 (465)	0.69 (0.55-0.87)^f^	0.78 (0.58-1.05)
HIV test in the past 12 months			
Yes	55.3 (679)	1.00	1.00
no	61.1 (834)	1.28 (1.08-1.48)^f^	1.36 (1.12-1.65)^f^
UVI^b^			
Yes	61.9 (208)	1.16 (0.94-1.50)	
No	57.8 (1311)	1	-
Coverage of HIV intervention			
Yes	57.0 (1186)	1.00	1.00
no	63.8 (333)	1.33 (1.09-1.62)^f^	1.21 (0.94-1.56)
Number of male partners in the past 6 months			
1	59.3 (463)	1.00	1.00
2	63.3 (394)	1.18 (0.96-1.47)	1.27 (1.02-1.59)^e^
3	67.4 (302)	1.42 (1.11-1.81)^f^	1.49 (1.16-1.92)^f^
4-9	71.6 (272)	1.73 (1.33-2.25)^f^	1.77 (1.35-2.33)^f^
≥10	73.5 (86)	1.91 (1.23-2.94)^f^	1.85 (1.18-2.91)^f^

^a^Only factors significant in univariate analysis were included in the multivariate logistic regression model.

^b^Unprotected anal intercourse.

^c^Univariate logistic regression analysis; ^d^Multivariate logistic regression analysis.

^e^ *P* < 0.05; ^f^ *P* < 0.01.

The crude and adjusted model also indicated that compared with those who were single, the participants who were cohabiting with male partner, divorced or widowed had significantly higher odds of engaging in UAI, with AORs of 2.15 (95% CI: 1.66-2.79) and 2.80 (95% CI: 1.54-5.07), respectively. Table 2 also pointed out that those who did not test for HIV in the past year (AOR = 1.36, 95% CI: 1.12-1.65) or did not receive any HIV related services (AOR = 1.21, 95% CI: 0.94-1.56) had higher odds of engaging in UAI, despite the latter association being not significant in the adjusted model. In addition, the risk of having UAI increased with the increasing number of male partners in the past six months. For example, the odds of engaging in UAI was 85% higher for those who had 10 or more partners in the past six months (OR: 1.85, 95% CI: 1.18-2.91) compared to those who had only one partner in the past six months.

The results of our study indicated that the age of first sex with male did not correlate with UAI. The stratified trend analysis (Table 3) demonstrated that the proportions of UAI in different stratums were stable during the study period, and the P-value were greater than 0.05. For example, UAI was stable among the participants who were aged between 20–29, single, and attended college or above, barring slight fluctuations.

**Table 3:** **Characteristics correlated with unprotected anal intercourse among MSM in Guangzhou: trend from 2008 to 2013** ^***a***^

Characteristics	Percentage of UAI in different stratums							
	2008	2009	2010	2011	2012	2013	***χ*** ^2^value	***P*** ^***b***^
	% (n)	% (n)	% (n)	% (n)	% (n)	% (n)		
Age (years)								
<20	64.3 (9)	70.0 (7)	69.2 (9)	80.0 (12)	88.9 (8)	73.3 (1)	0.94	0.333
20-29	60.8 (132)	55.1 (125)	61.2 (150)	62.4 (156)	55.7 (127)	58.7 (226)	0.07	0.786
30-39	64.0 (71)	54.9 (62)	58.3 (70)	67.3 (68)	55.5 (71)	51.7 (90)	2.61	0.106
≥40	56.8 (21)	45.7 (16)	40.7 (11)	35.3 (12)	58.3 (21)	57.6 (34)	0.55	0.458
Marital status								
Single	58.4 (125)	47.8 (111)	57.0 (146)	60.8 (158)	53.5 (138)	52.3 (225)	0.37	0.544
Cohabiting with male partner	73.1 (49)	69.6 (48)	64.5 (40)	76.1 (35)	68.3 (43)	77.9 (81)	0.88	0.349
Married	58.2 (46)	56.5 (39)	63.5 (47)	56.2 (50)	53.6 (37)	52.6 (40)	0.84	0.360
Divorced or widowed	68.4 (13)	80.0 (12)	53.8 (7)	100.0 (5)	81.8 (9)	65.2 (15)	0.01	0.952
Education level								
Junior high school or lower	59.7 (43)	66.0 (31)	62.1 (41)	62.3 (33)	61.2 (30)	74.3 (26)	0.80	0.372
Senior high school	62.2 (69)	55.6 (55)	64.3 (74)	65.0 (91)	59.2 (61)	59.1 (65)	0.01	0.914
College or higher	61.7 (121)	51.9 (124)	55.8 (125)	59.9 (124)	54.6 (136)	55.3 (270)	0.44	0.509
Number of male partners in the past 6 months								
1	69.1 (65)	58.5 (72)	57.0 (69)	58.5 (72)	55.1 (75)	59.8 (110)	1.31	0.252
2	60.0 (48)	58.7 (54)	59.4 (57)	70.4 (69)	68.0 (66)	62.9 (100)	1.25	0.263
3	69.6 (48)	65.2 (43)	77.8 (49)	72.7 (48)	66.7 (48)	58.9 (66)	2.39	0.122
4-9	75.4 (52)	60.5 (23)	80.0 (48)	70.6 (48)	62.5 (25)	72.4 (76)	0.16	0.689
≥10	83.3 (20)	62.1 (18)	85.0 (17)	78.6 (11)	72.2 (13)	58.3 (7)	0.07	0.403
HIV test in the past 12 months								
Yes	64.5 (40)	53.3 (146)	55.1 (98)	55.4 (98)	59.1 (117)	53.3 (180)	0.17	0.679
No	60.9 (193	57.7 (64)	62.6 (142)	67.1 (147)	54.3 (107)	61.4 (181)	0.03	0.863

^a^Only significant factors in multivariate logistic regression were analyzed.

^b^Linear-by-linear association chi-square test.

## Discussion

In this serial cross-sectional study conducted in Guangzhou, China, we found a high proportion of MSM engaged in UAI in each year, and this proportion did not change significantly in the past six years. We further found that younger age, lower education, cohabiting with male partner, widowed or divorced, not tested for HIV in the last year, and having more than one male partner in the past six months were associated with UAI. These findings were very important for the design of targeting intervention strategies, in order to bring down the high rate of UAI, and in turn, reduce the chance of HIV and other STIs transmission.

Our study indicated that the overall UAI rate was high in Guangzhou, and this result is almost the same as one study conducted by our team in 2008 [13]. Similar results were also reported in several other cities in China. For example, Tang W et al. reported that the UAI rate was 62.3% in Nanjing at the year of 2008 [14]. Feng L reported that the UAI rate was about 62% in Chongqing in 2010 [15]. Recently, an updated meta-analysis summarized that the overall UAI rate among Chinese MSM was about 53% (95% CI: 51–56%) [16], which is lower than the overall proportion of UAI in Guangzhou.

Besides this, our study also found that the UAI proportion was consistently high during the study period, which indicated that the intervention implemented in the past few years did not bring down the high-risk behaviors of the MSM in Guangzhou. This may explain why the HIV prevalence consistently increased in Guangzhou during 2008 and 2013 (Increased from 5.2% in 2008 [13] to 11.4% in 2013 [8]). Thus, an effective intervention method that can change the behaviors of MSM is urgently needed since UAI is a well-known risk factor of HIV and other STIs [17]-[19].

Given the high HIV prevalence and incidence (about 10/100 person-years, unpublished data) among MSM in Guangzhou, the consistent high UAI rate is an important signal to us. This consistent high risk behavior may further fast the spreading of HIV transmission among MSM in Guangzhou. Effective intervention programs targeting this high risk behavior are urgently needed. Risk-reduction-counseling, which is considered to be an effective intervention method, that could bring down the risk behaviors of MSM should be adapted and used. In addition, other intervention methods include but not limited to condom promotion and distribution, internet-facilitated interactive intervention (developed by our study team, unpublished data) and HIV screening also should be implemented.

The results of our study also indicated that 18% of the participants were married, and about 13% of the participants engaged in UVI in the past six month. Given the higher HIV prevalence in MSM in Guangzhou (11.4% in 2013), HIV prevention strategies that prevent the HIV transmission from MSM to their female partners are urgently needed.

Our study found that not being tested for HIV in the past year was associated with UAI. This finding is similar with the result of one study conducted in Scotland [20]. However, this is first study according to our knowledge that reported the correlation between UAI and HIV testing behaviors in China. In addition, lack of HIV related intervention was also positive correlated with UAI. These results indicated that the increasing HIV testing and related intervention could potentially bring down the proportion of UAI in MSM in Guangzhou. In the meantime, HIV testing not only increased the detection ratio of HIV infection, but also provided an opportunity for implementing positive intervention techniques such as serosorting and strategic positioning, which can further reduce the second transmission.

Our study found that younger age, particular for those who aged less than 20, were associated with UAI. Similar result was reported by in San Francisco [21]. One potential reason is that young people may lack of the knowledge of self-protection, and more likely to take risks in the pursuit of excitement. Similar to the findings of a study conducted in Jinan [22], our results too indicated that participants who had multiple sexual partners in the past six month also had higher odds of engaging in UAI. Thus, it is important to consider this evidence in the design of future intervention programs by specifically target in reducing the number of partners.

The results of our study pointed out that cohabiting with male partner was associated with UAI. One possible explanation for this phenomenon is that the cohabited MSM are more likely to have stable or regular partners, and they already established mutual trust with each other, and more likely not to use condoms. Similar results were found for divorced or widowed participants. In addition, our results corroborated earlier studies indicating that poor education is significantly associated with UAI [22]. One possible explanation for this situation is that the participants who received less education may have a poor understanding of health and disease, and therefore are generally do not take precautionary measures [23].

In our study, we observed some interesting findings, for example, less than one third were residents of Guangzhou, approximately one third did not identify as homosexual and the association with increased partner numbers and UAI. These indicated that the proportion of participants who had casual partners may high in Guangzhou. Given the high UAI rate and high HIV prevalence among MSM in Guangzhou, this may spread the transmission of HIV in the future. We should take these phenomena into consideration in our future study design and intervention effort.

The strength of our study was the use of serial cross-sectional study design, larger sample size and the longer observational time (six years). As an observational study, our study has certain limitations. First, we did not collect the information on the UAI with different partnerships, thus prevented exploration the factors under different relationship. We also did not differentiate unprotected safer sex practices such as serosorting, strategic positioning and withdrawal before ejaculation from UAI, which may limited our ability to understand the current drivers of HIV transmission in the community. Second, despite of using same sampling method during the study period, we still found that the participants were marginally incomparable. For example, some demographic characteristics of the participants were waned year by year. Third, since this is a cross-sectional study design, lacked of temporality prevents us from drawing any causal inferences. Fourth, all of the information collected in our study was based on the self-report, and possible information bias may exist in our study, due to the problem of social desirability. Fifth, we did not collect information on the proportion of the participants who attended the study for more than once, which limited our ability to assess the possible effect of intervention program that we implemented in our study. Moreover, selection bias can also be a potential threat to validity of current reported study, since we did not collect information on the response rate of the eligible participants.

## Conclusion

In conclusion, we found that the proportion of MSM engaged in UAI was consistently high during every year of the study period. Also, younger age, poor education, cohabiting with male partner, and widowed or divorced participants had higher odds of engaging in UAI. More importantly, not being tested for HIV in the past year and having multiple male partners in the past six months were significantly correlated with UAI. Effective intervention strategies are urgently needed to bring down the risk behaviors of the MSM in Guangzhou, in order to control HIV/STIs epidemic in this specific population.
